# HIF-2α Depletion and HIF-1α Overexpression in Vulnerable Brain Regions Distinguish Alzheimer’s Disease with Cerebral Amyloid Angiopathy

**DOI:** 10.3390/ijms27177784

**Published:** 2026-08-31

**Authors:** Vladimir S. Sukhorukov, Tatiana I. Baranich, Olga V. Velts, Kseniia M. Okulova, Dmitry N. Voronkov, Ekaterina V. Shcherbak, Anna V. Egorova, Natalia M. Mudzhiri, Dmitry S. Lazarev, Alexander P. Raksha, Alexander N. Yatskovskiy, Valeria V. Glinkina, Michail A. Piradov

**Affiliations:** 1 Russian Center of Neurology and Neurosciences, Laboratory of Neuromorphology, 125367 Moscow, Russia; vsukhorukov@gmail.com (V.S.S.);; 2 Department of Anatomy and Histology, I.M. Sechenov First Moscow State Medical University (Sechenov University), 125009 Moscow, Russia; 3 Pirogov City Clinical Hospital No. 1, 119049 Moscow, Russia; 4 Department of Pathology, Pirogov Russian National Research Medical University, 117513 Moscow, Russia

**Keywords:** hippocampus, hypoxia, anterior cingulate cortex, Alzheimer’s disease, cerebral amyloid angiopathy, aging

## Abstract

Hypoxia-inducible factors (HIF-1α, HIF-2α, HIF-3α) regulate cellular adaptation to oxygen deprivation, but their region-specific roles in Alzheimer’s disease (AD) and AD with cerebral amyloid angiopathy (CAA) remain unclear. Using post-mortem human brain tissue from aging, AD, and AD + CAA groups, we measured all three HIF isoforms in hippocampal subfields (CA1, CA2, CA4, dentate gyrus) and anterior cingulate cortex (ACC) layers 3 and 5. In the AD hippocampus, two distinct patterns emerged: ischemia-resistant regions (CA4, DG) maintained HIF-2α and showed relative resilience, whereas vulnerable regions (CA1, CA2) exhibited HIF-1α upregulation, HIF-3α loss, and HIF-2α dysregulation. The ACC contrasts sharply with the hippocampus by preserving coordinated HIF-1α/HIF-3α regulation during aging and AD, with layer-specific divergence (exhaustion in layer 3 vs. resilience in layer 5) emerging only upon addition of CAA. Notably, HIF-2α in ACC neurons remains stably elevated across all conditions. Taken together, our results highlight HIF-2α as a potential contributor to regional vulnerability and raise the possibility that maintaining HIF-2α levels, in addition to or instead of modulating HIF-1α, could be worthy of further investigation in the context of AD and related vascular changes.

## 1. Introduction

Alzheimer’s disease (AD) is the leading cause of dementia worldwide, characterized by progressive memory loss, cognitive decline, and ultimately disability and death. Pathomorphologically, the disease is marked by extracellular β-amyloid (Aβ) plaques, intracellular neurofibrillary tangles composed of hyperphosphorylated tau protein, as well as neuroinflammation and selective loss of synapses and neurons [1]. Given the exponential increase in AD prevalence with rising life expectancy, targeted therapies for this disease have become a pressing priority.

The hippocampus, which plays a central role in learning and episodic memory, is one of the earliest and most severely affected structures in AD [2]. Hippocampal atrophy and disrupted connectivity with other brain regions are already observed at early disease stages, with the CA1 field showing more pronounced neurodegeneration and glial activation than other subfields [3]. As AD progresses, the CA1–CA4 regions and the dentate gyrus atrophy in a staged manner, and the degree of structural damage directly reflects the level of cognitive impairment [4]. In early and mild cognitive impairment, the subiculum and CA1 are primarily affected, whereas in severe dementia, pathology extends to other hippocampal subfields [4,5]. Alongside the hippocampus, the anterior cingulate cortex (ACC) is a key center for cognitive control of emotion, belonging to the limbic system and closely interacting with the hippocampus. Magnetic resonance imaging (MRI) studies have shown significant atrophy of the rostral and caudal portions of the cingulate gyrus in AD [6]. Moreover, ACC functional activity is altered already at the stage of mild cognitive impairment, correlating with deficits in episodic memory and emotion regulation [7]. Of particular note, ACC neurons undergo abnormal changes at early AD stages, directly correlating with depression and loss of insight in patients [8]. Thus, investigation of neurons in both the hippocampus and the anterior cingulate cortex is essential for in-depth understanding of the mechanisms underlying memory impairment and cognitive-affective disturbances in AD.

A key factor linking age-related changes (including vascular) and abnormal neurodegeneration is ischemia–hypoxia. Hypoxia substantially contributes to metabolic disturbances, oxidative stress, and neuronal death in regions vulnerable to AD. The hypoxic component becomes particularly relevant when comparing two common, often coexisting forms of pathology: classic Alzheimer’s disease (AD) and AD associated with cerebral amyloid angiopathy (CAA). The latter is characterized by amyloid deposition in the walls of leptomeningeal and intracortical cerebral vessels, leading to chronic hypoperfusion, impaired cerebrovascular autoregulation, and consequent aggravation of ischemic changes in neural tissue. Moreover, AD with CAA exhibits a higher total amyloid burden, which may potentiate the direct effects of β-amyloid on cellular signaling pathways, including HIF-mediated hypoxic responses.

The central molecular regulators of adaptation to oxygen deprivation are hypoxia-inducible factors (HIFs). β-amyloid has been shown to directly modulate HIF expression and stability independently of oxygen levels: Aβ directly induces HIF-1α expression in vitro, and high amyloid levels characteristic of advanced pathology may exacerbate dysregulation of HIF-dependent genes [9]. HIF-1α regulates genes involved in angiogenesis (VEGF), glucose transport (GLUT1/3), glycolysis, erythropoiesis (EPO), and cell survival and may exert neuroprotective effects by maintaining energy metabolism [9,10]. However, in AD, HIF-1α plays a dual role: on the one hand, it promotes amyloidogenic APP processing through BACE1 activation and also enhances neuroinflammation and insulin resistance; on the other hand, under certain conditions (e.g., low Aβ levels or preconditioning), it protects neurons from Aβ toxicity and tau hyperphosphorylation [10,11]. Unlike HIF-1α, HIF-2α is stabilized in the adult mouse brain even under normoxia conditions, reflecting its critical role in neurogenesis, neuronal migration, and synaptic plasticity [12]. HIF-2α directly regulates antioxidant enzymes, including superoxide dismutase 2 (SOD2), as well as neuroprotective erythropoietin. Notably, marked upregulation of HIF-2α in hippocampal pericytes and neurons in patients with amyloid plaques but preserved cognitive status points to a key neuroprotective role of this factor [13,14]. The third isoform, HIF-3α, has traditionally been considered a dominant-negative regulator; however, its precise role in AD neurons remains unclear. In AD astrocytes, HIF-3α enhances oxidative stress and mitochondrial dysfunction through transcriptional activation of TXNIP, thereby promoting disease progression [15]. Furthermore, in APP/PS1 double-transgenic mice with disrupted circadian rhythm, a significant increase in HIF3A expression in brain tissue was observed compared to controls [16]. In vitro experiments in human cells and in vivo studies in rodents have established that HIF-1α and HIF-2α, both individually and together, induce HIF-3α expression [17]. Thus, the HIF-mediated response in AD is characterized by complex cross-regulation of subunits, underscoring the need to study not only HIF-1α but also HIF-2α and HIF-3α expression, about which much less is known. However, a systematic comparison of all three HIF isoforms in different hippocampal subfields and the anterior cingulate cortex in classic AD and AD with CAA has not yet been performed.

This study comprehensively analyzed the abundance and distribution of all three HIF isoforms (HIF-1α, HIF-2α, HIF-3α) in neurons of various hippocampal subfields (CA1, CA2, CA4, dentate gyrus) and the anterior cingulate cortex using autopsy material from a physiological aging group and AD patients divided into two groups: AD without CAA and AD with CAA (AD + CAA). Comparative analysis of these groups is of interest not only due to differences in the severity of hypoxia but also because of the distinct amyloid burden: high Aβ concentrations in the vascular wall in CAA may modulate HIF-mediated signaling pathways, shifting the balance between neuroprotection and abnormal processes in vulnerable brain regions.

## 2. Results

### 2.1. CA1 Region

During aging, the levels of both nuclear and cytoplasmic HIF-1α did not differ from those in the young group. In contrast, HIF-2α content increased in both nuclei and cytoplasm, whereas expression of both HIF-3α fractions decreased.

In AD, HIF-1α increased in both nuclei and cytoplasm, as did the cytoplasmic fraction of HIF-2α, while the nuclear pool of HIF-2α did not differ from the aging group. HIF-3α expression continued to decline in both fractions compared to the aging group.

**Figure 1:**
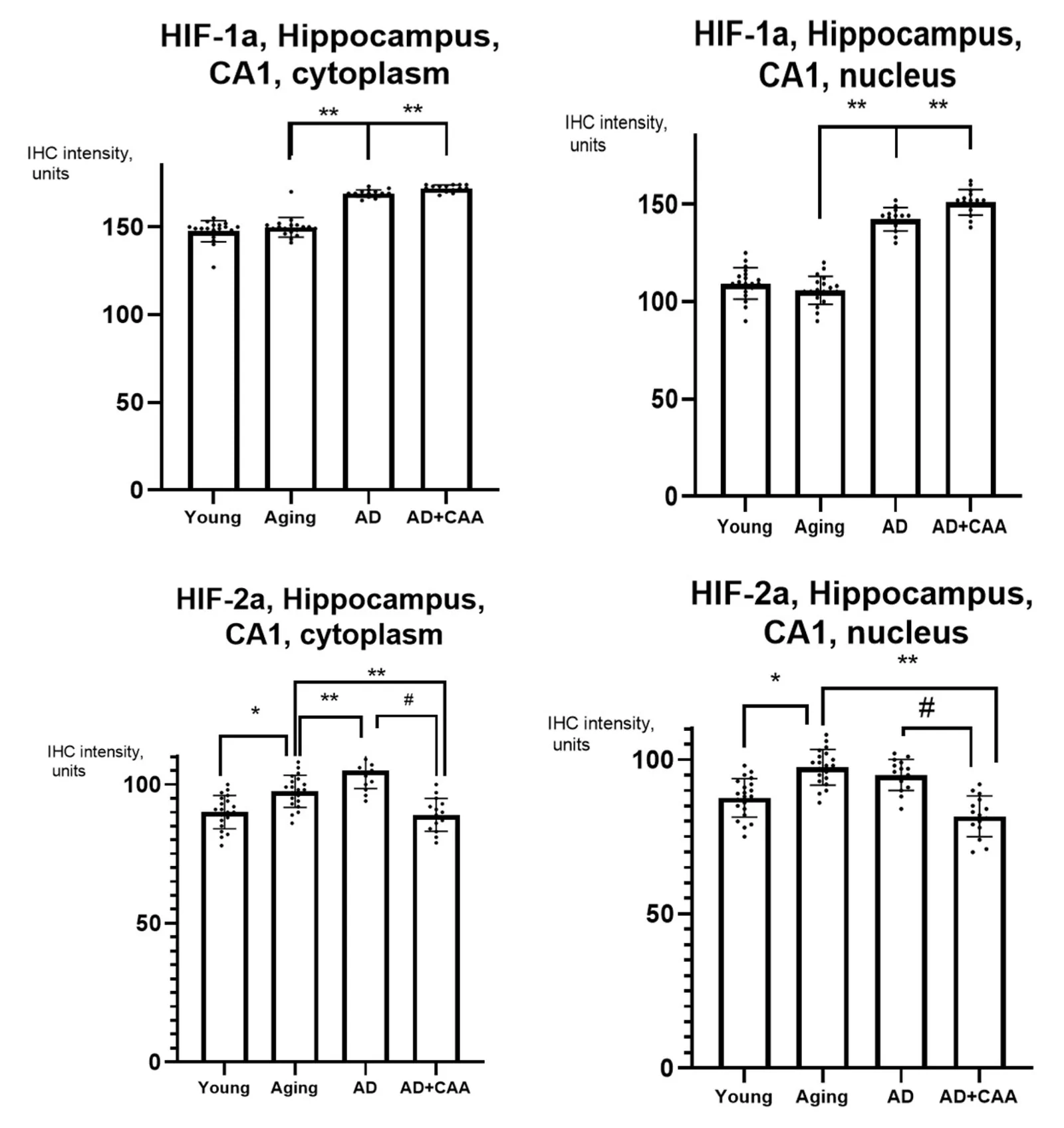

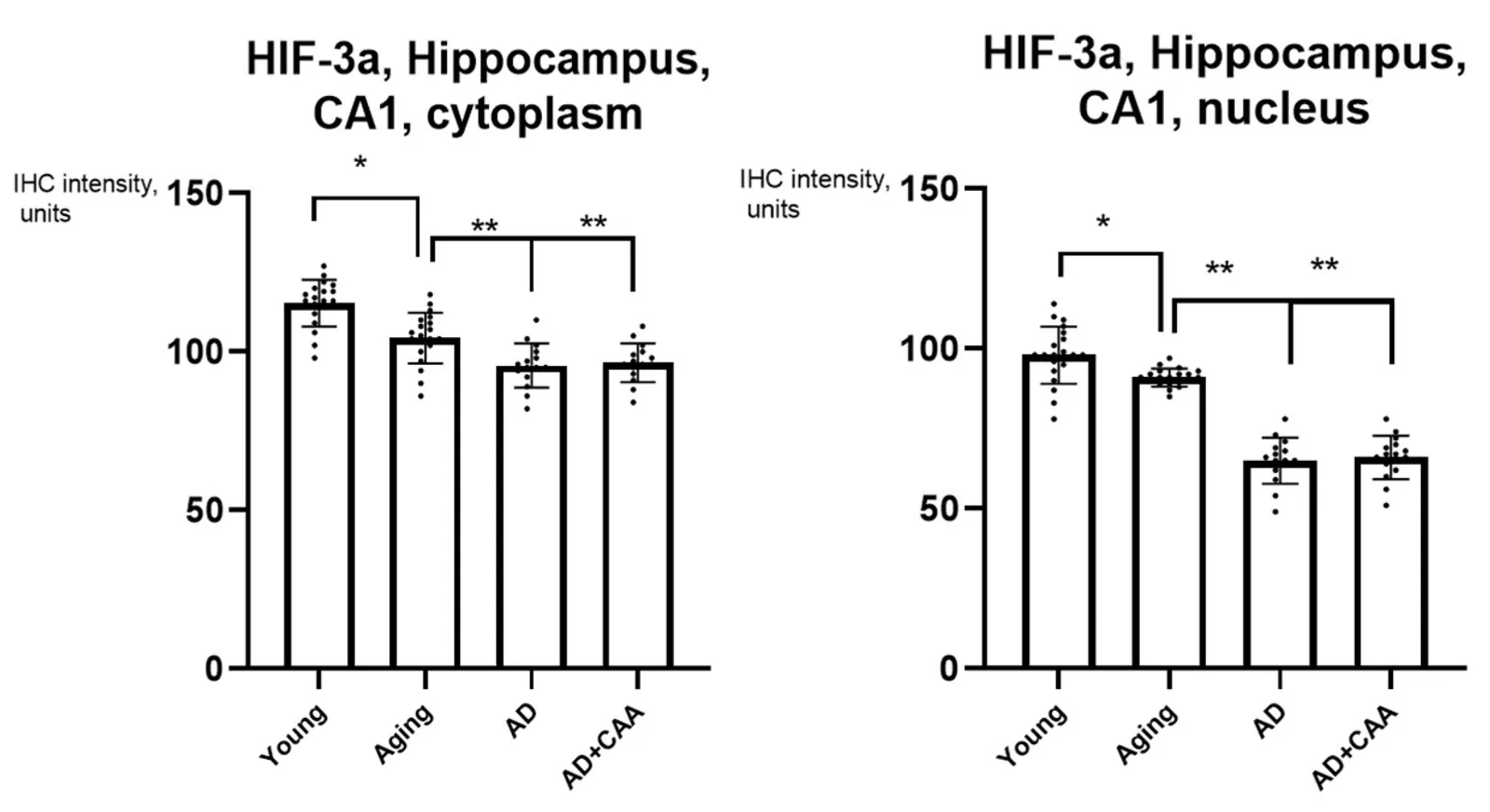
Results of morphometric analysis for immunohistochemical distribution of HIF-1α, HIF-2α, HIF-3α markers in neurons of CA1 region of hippocampus. Asterisks indicate statistically significant differences: *—from young group; **—from aging group; #—from AD group. Y axis—level of IHC intensity ± SD. Each dot indicates one individual sample.

In AD with CAA, the HIF-1α and HIF-3α profiles mirrored those of classic AD (increase and decrease, respectively). However, HIF-2α levels in both nuclei and cytoplasm were significantly lower than in classic AD (Figure 1).

### 2.2. CA2 Region

During aging, the nuclear fraction of HIF-1α decreased, while its cytoplasmic level remained comparable to that in the young group. The content of both HIF-2α fractions increased, whereas HIF-3α expression in both nuclei and cytoplasm decreased compared to the young group.

In AD, the levels of both HIF-1α fractions were elevated. In contrast to HIF-1α, expression of HIF-2α and HIF-3α in both nuclei and cytoplasm of CA2 neurons was lower than in the aging group.

**Figure 2:**
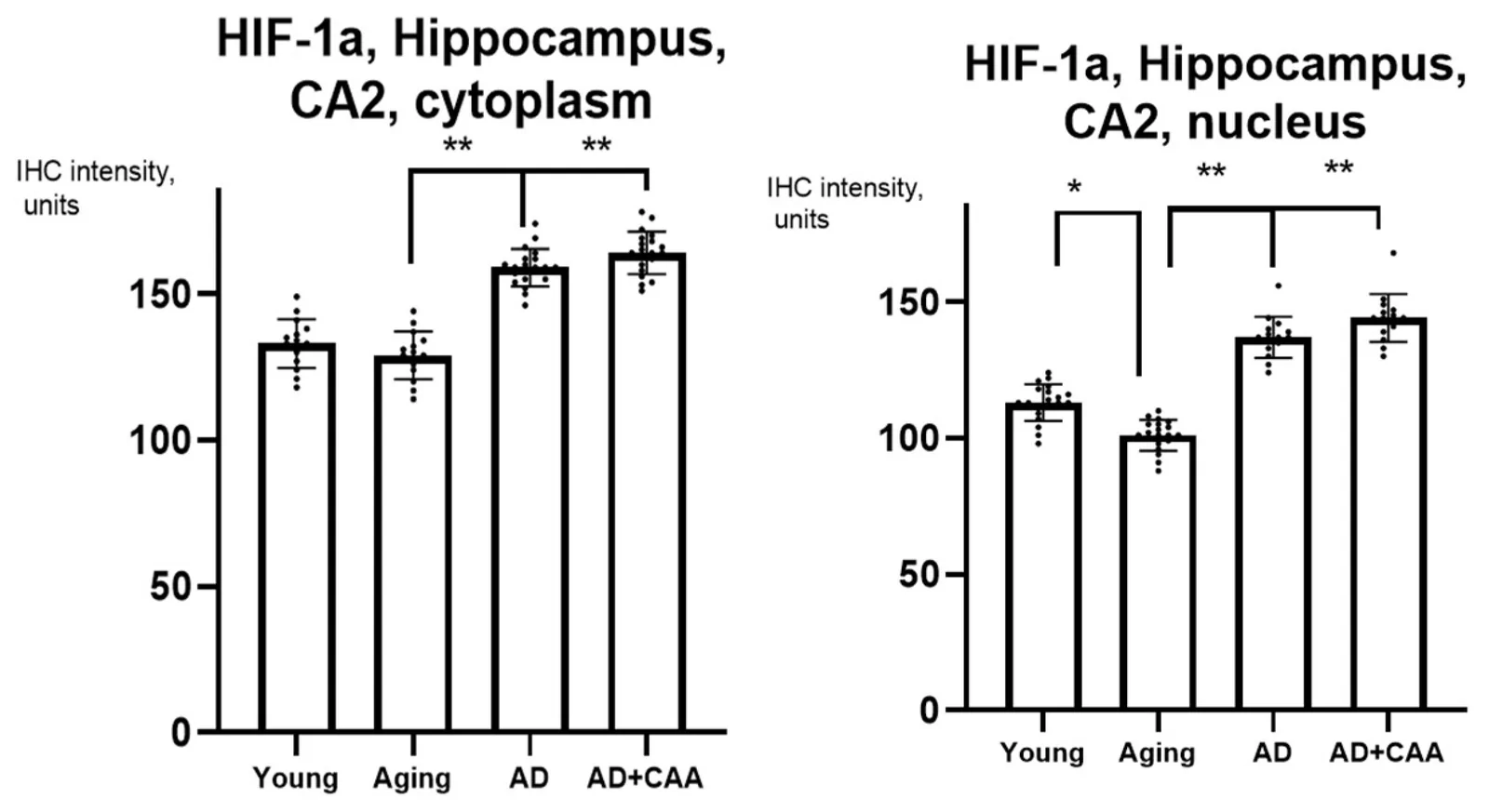

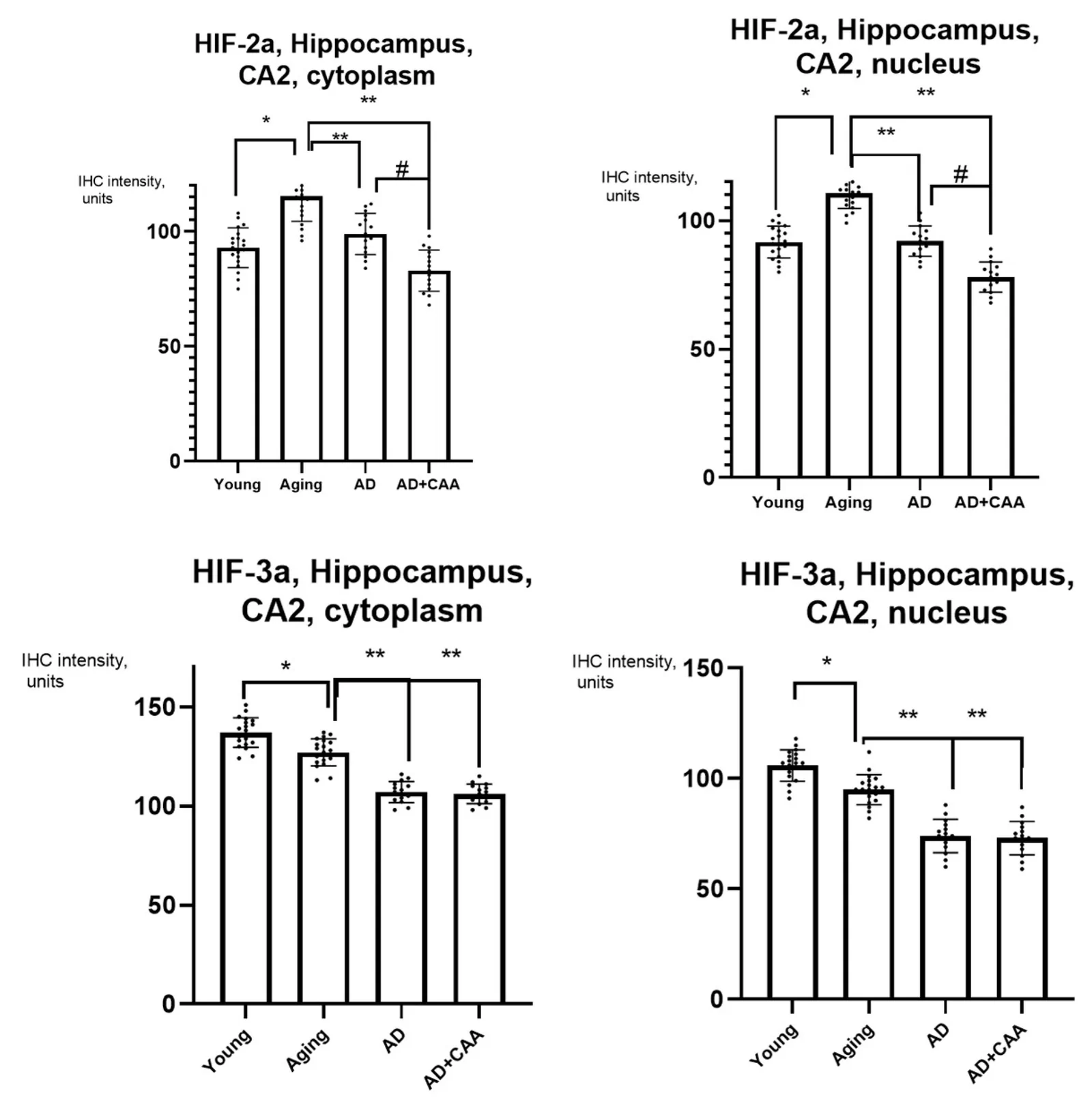
Results of morphometric analysis for immunohistochemical distribution of HIF-1α, HIF-2α, HIF-3α markers in neurons of CA2 region of hippocampus. Asterisks indicate statistically significant differences: *—from young group; **—from aging group; #—from AD group. Y axis—level of IHC intensity ± SD. Each dot indicates one individual sample.

In AD with CAA, the HIF-1α profile corresponded to that of classic AD (increase in both fractions compared to the aging group). HIF-3α expression also mirrored the changes characteristic of AD (decrease in nuclei and cytoplasm). However, the levels of both HIF-2α fractions were further reduced compared not only to the aging group but also to AD (Figure 2).

### 2.3. CA4 Region

During aging, expression of all three HIF subunits—HIF-1α, HIF-2α, and HIF-3α—increased in both nuclei and cytoplasm compared to the young group.

**Figure 3:**
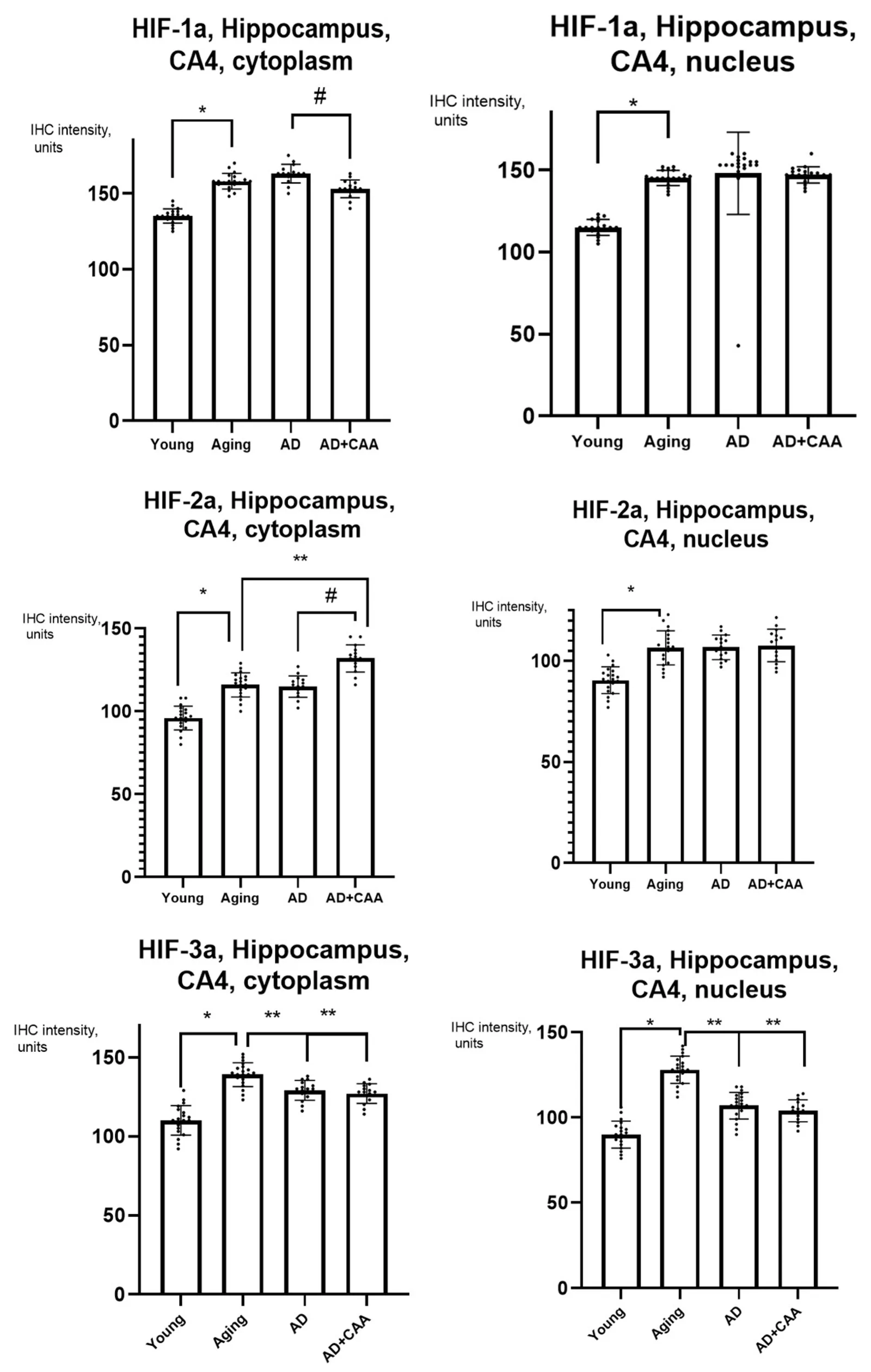
Results of morphometric analysis for immunohistochemical distribution of HIF-1α, HIF-2α, HIF-3α markers in neurons of CA4 region of hippocampus. Asterisks indicate statistically significant differences: *—from young group; **—from aging group; #—from AD group. Y axis—level of IHC intensity ± SD. Each dot indicates one individual sample.

In AD, the levels of HIF-1α and HIF-2α in nuclei and cytoplasm remained at the aging group level. In contrast, HIF-3α expression in both fractions was decreased compared to the aging group (Figure 3).

**Figure 4:**
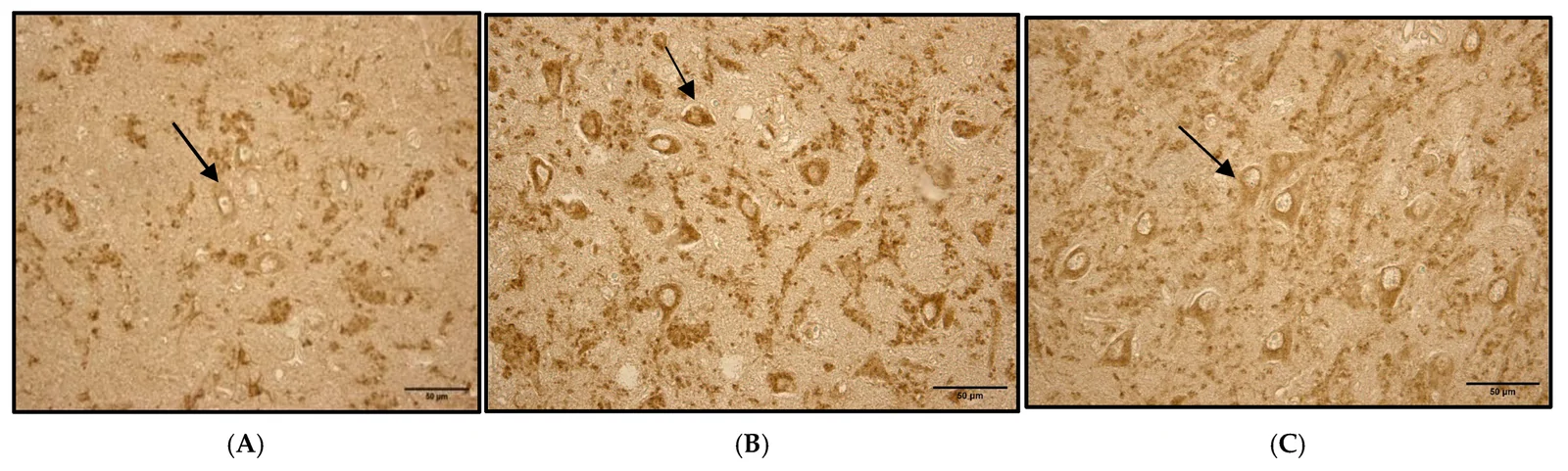
(**A**–**C**) Immunohistochemical distribution of HIF-1α marker in neurons of CA4 region of hippocampus (arrows). (**A**) Staining in aging group neurons; (**B**) staining in AD patient neurons; (**C**) staining in AD with CAA patient neurons. Objective magnification: 40×. Scale bar = 50 μm.

In AD with CAA, the nuclear level of HIF-1α did not differ from values in either the aging group or classic AD, whereas its cytoplasmic fraction was comparable to aging but lower than in classic AD (Figure 4). HIF-2α content showed changes exclusively in the cytoplasm, where it exceeded values in both aging and classic AD. Expression of both HIF-3α fractions mirrored the pattern observed in classic AD, remaining reduced relative to the aging group.

### 2.4. Dentate Gyrus

During aging, expression of all three HIF subunits—HIF-1α, HIF-2α, and HIF-3α—increased in both nuclei and cytoplasm of neurons compared to the young group.

**Figure 5:**
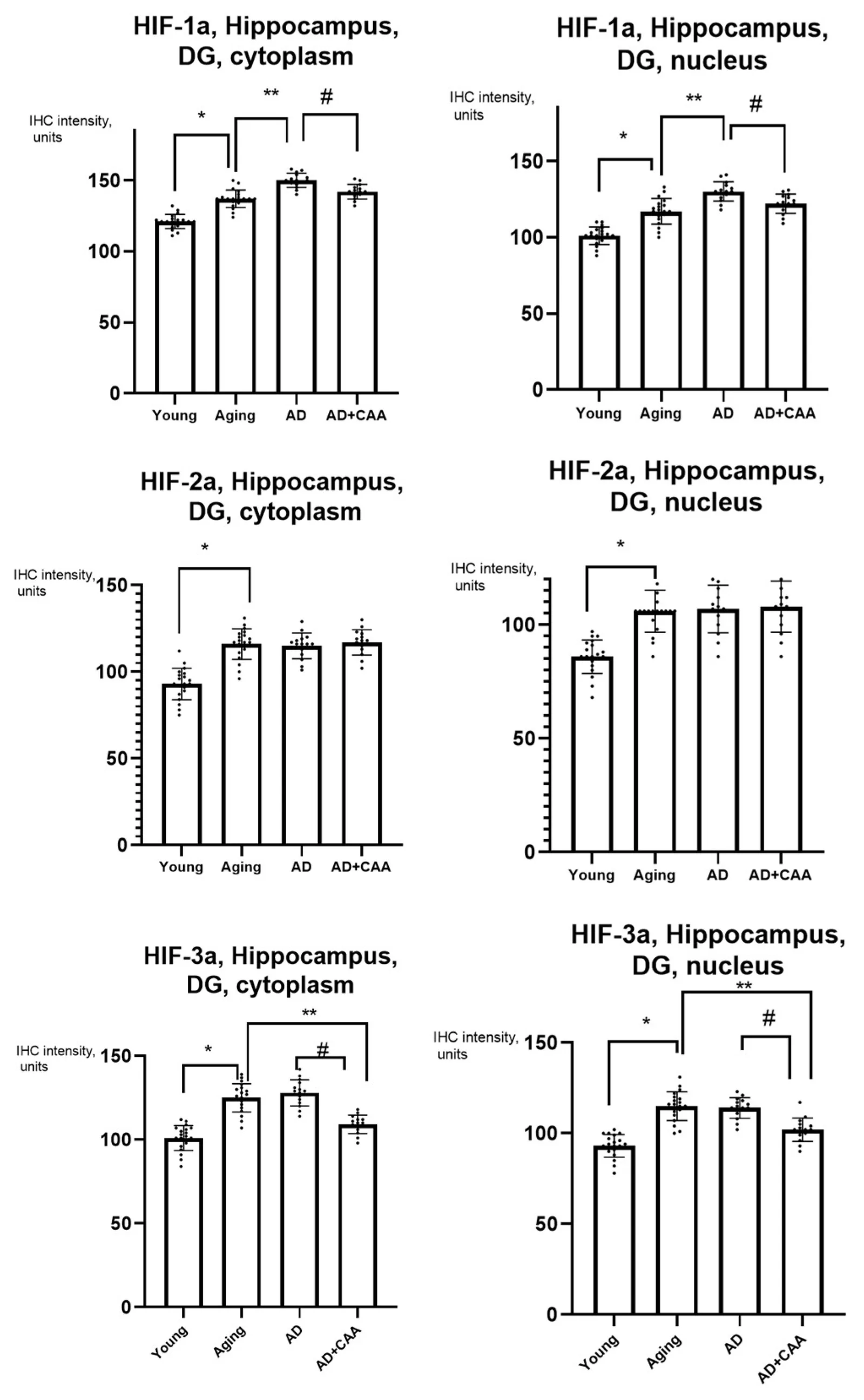
Results of morphometric analysis for immunohistochemical distribution of HIF-1α, HIF-2α, HIF-3α markers in neurons of DG region of hippocampus. Asterisks indicate statistically significant differences: *—from young group; **—from aging group; #—from AD group. Y axis—level of IHC intensity ± SD. Each dot indicates one individual sample.

In AD, HIF-1α levels in both nuclei and cytoplasm increased compared to the aging group, whereas expression of both HIF-2α and HIF-3α fractions remained at levels comparable to the aging group (Figure 5).

**Figure 6:**
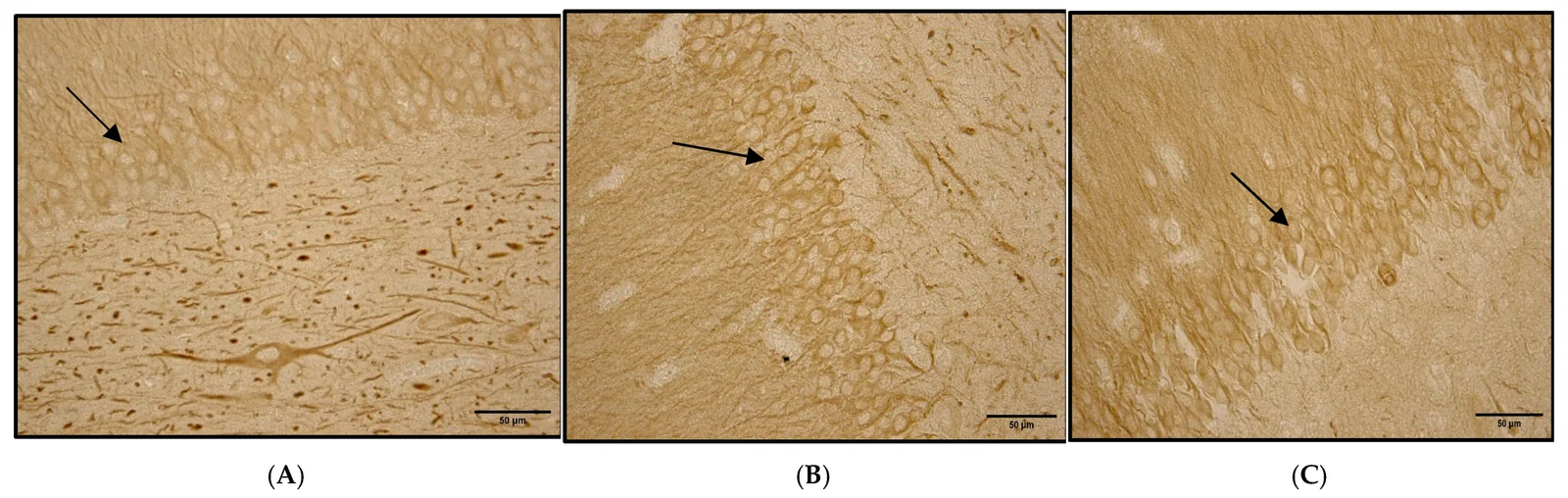
(**A**–**C**) Immunohistochemical distribution of HIF-2α marker in neurons of DG region of hippocampus (arrows). (**A**) Staining in young group neurons; (**B**) staining in aging group neurons; (**C**) staining in AD patient neurons. Objective magnification: 40×. Scale bar = 50 μm.

In AD with CAA, the profile of both HIF-1α fractions was comparable to the aging group but lower than in classic AD. In contrast to HIF-1α, HIF-2α levels in nuclei and cytoplasm did not differ from values in either the aging group or the AD group (Figure 6). Expression of both HIF-3α fractions decreased compared to both comparison groups—aging and AD.

### 2.5. Anterior Cingulate Cortex

**Figure 7:**
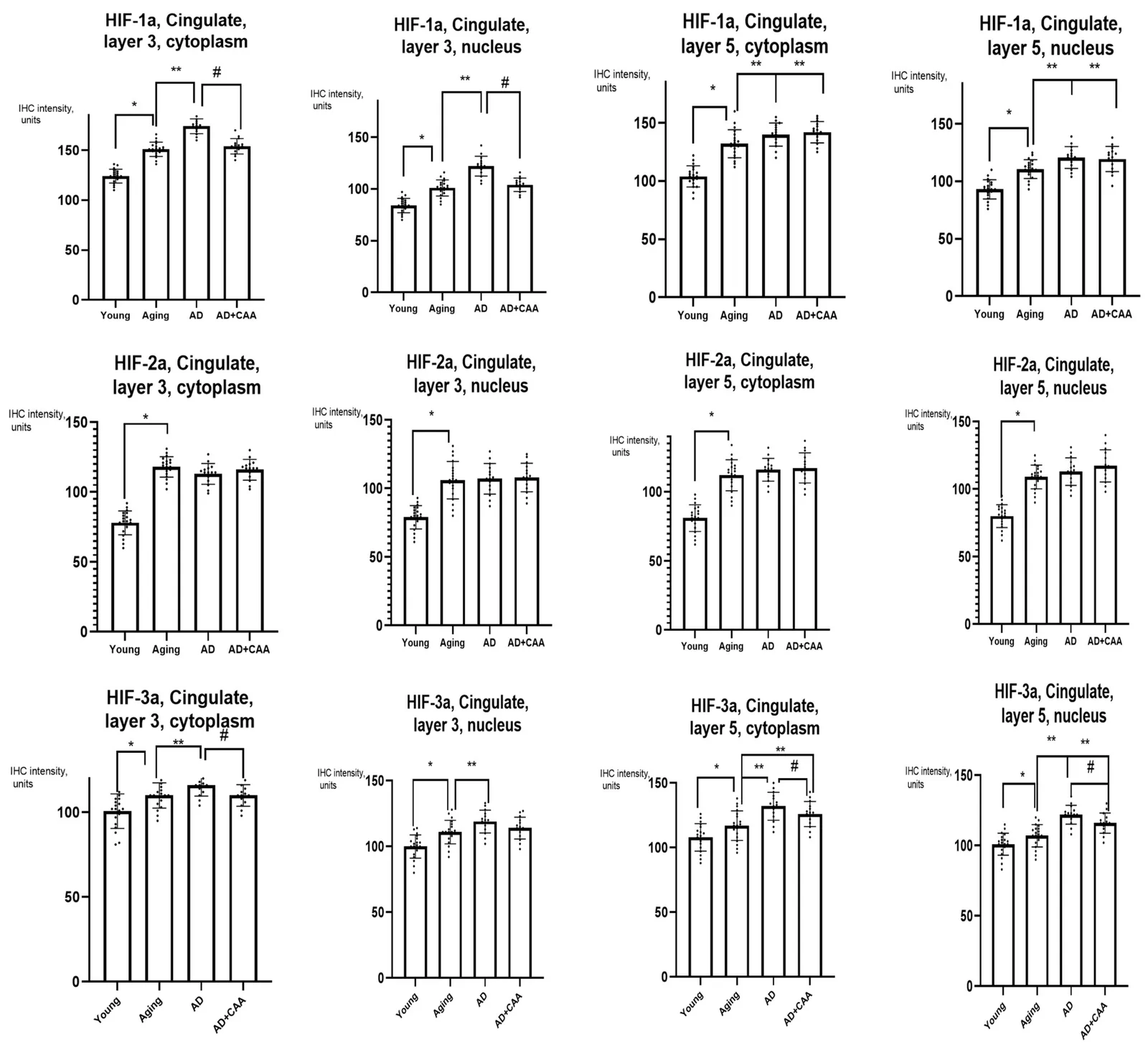
Results of morphometric analysis for immunohistochemical distribution of HIF-1α, HIF-2α, HIF-3α markers in neurons of anterior cingulate cortex. Asterisks indicate statistically significant differences: *—from young group; **—from aging group; #—from AD group. Y axis—level of IHC intensity ± SD. Each dot indicates one individual sample.

During aging, expression of all three HIF subunits—HIF-1α, HIF-2α, and HIF-3α—increased in both nuclei and cytoplasm of neurons in layers 3 and 5 compared to the young group (Figure 7).

**Figure 8:**
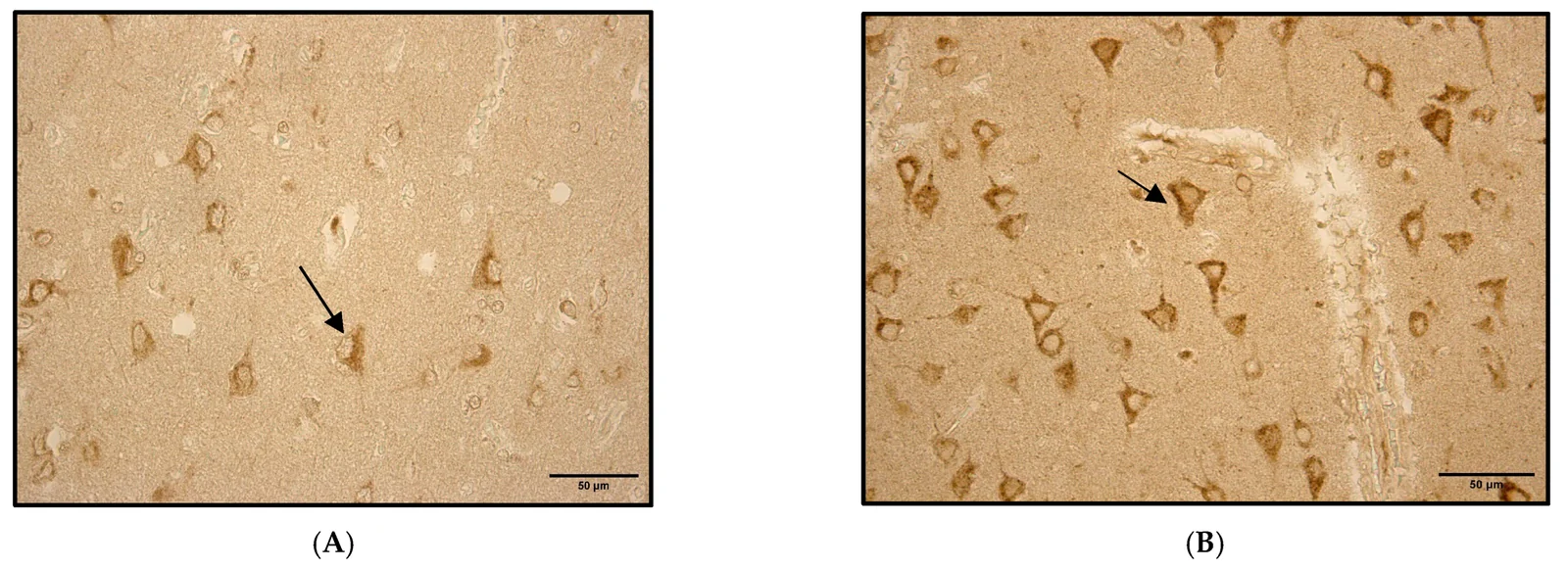
(**A**,**B**) Immunohistochemical distribution of HIF-1α marker in neurons of layer 5 of anterior cingulate cortex (arrows). (**A**) Staining in aging group neurons; (**B**) in AD patient neurons. Objective magnification: 40×. Scale bar = 50 μm.

**Figure 9:**
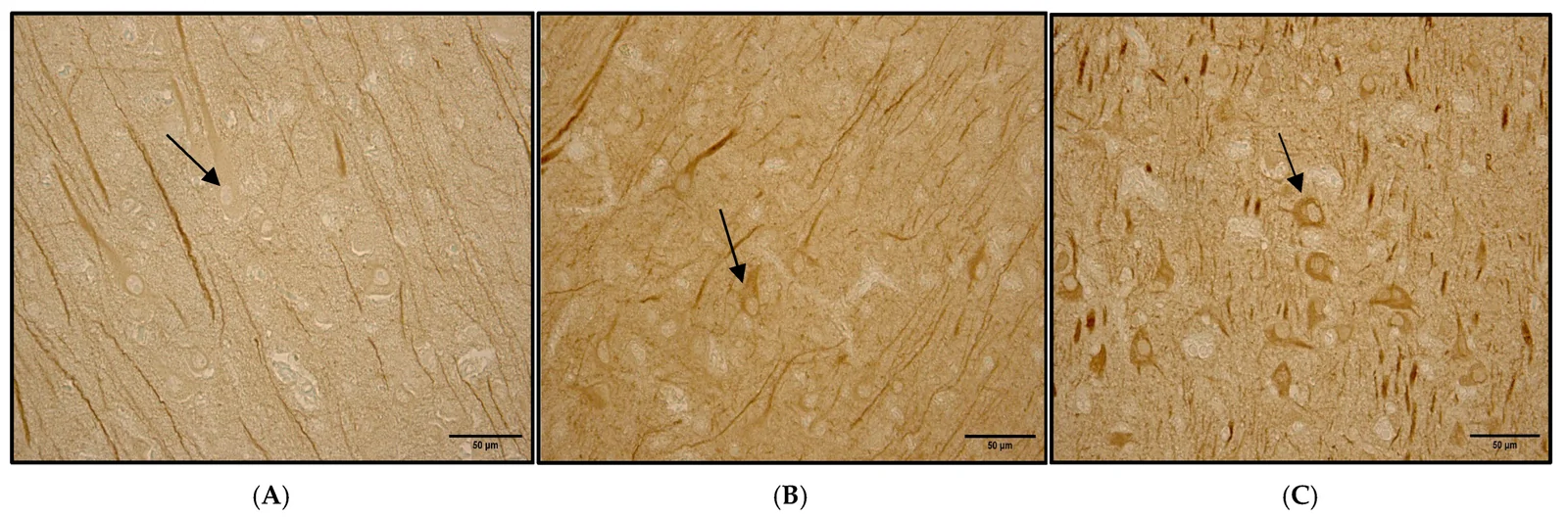
(**A**–**C**) Immunohistochemical distribution of HIF-2α marker in neurons of layer 5 of anterior cingulate cortex (arrows). (**A**) Staining in young group neurons; (**B**) staining in aging group neurons; (**C**) staining in AD patient neurons. Objective magnification: 40×. Scale bar = 50 μm.

In AD, HIF-1α and HIF-3α levels in both nuclei and cytoplasm of neurons in layers 3 and 5 increased compared to the aging group (Figure 8), whereas expression of both HIF-2α fractions remained at levels comparable to the aging group (Figure 9).

In AD with CAA, in layer 3 neurons, the levels of both HIF-1α and HIF-3α fractions were comparable to the aging group but lower than in AD. In layer 5 neurons, expression of both HIF-1α fractions exceeded aging group values while remaining at the level observed in AD. In contrast to HIF-1α, HIF-3α levels in both nuclei and cytoplasm of layer 5 neurons also increased compared to aging but were reduced compared to AD. Levels of both HIF-2α fractions did not differ from values in the aging and AD groups across all layers studied.

## 3. Discussion

Our results indicate that the HIF system response during aging and AD is highly region-specific. In the hippocampus, two fundamentally different patterns can be distinguished, corresponding to the known anatomical and functional differentiation of this structure.

### 3.1. CA4 Region and Dentate Gyrus

In the CA4 and dentate gyrus (DG) regions, which are part of the ischemia-resistant hippocampal zone [10], physiological aging is associated with a synchronous increase in all three HIF isoforms studied—HIF-1α, HIF-2α, and HIF-3α. This coordinated response suggests preserved regulatory balance and may reflect more effective adaptation to age-related hypoxic stress. The concomitant increase in the negative regulator HIF-3α may limit HIF-1α transcriptional activity, preventing its excessive activation and associated pathological effects [11,12].

In AD, these regions show fundamentally different changes: in CA4, HIF-1α levels do not change compared to aging, whereas in the DG they are significantly elevated. A common feature of both regions is the maintenance of HIF-2α at a stable high level. HIF-2α is known to regulate expression of antioxidant enzymes (SOD2) and neuroprotective erythropoietin, supporting mitochondrial homeostasis and reducing oxidative stress [13,14]. The preserved expression of HIF-2α in CA4 and DG neurons is consistent with the relative resistance of these regions to neurodegeneration in AD, suggesting a potential protective association that warrants functional investigation.

At the same time, in both regions, AD is associated with a decrease in HIF-3α (in CA4) or its maintenance at the aging level (in DG). This suggests that the dentate gyrus is at an earlier stage of HIF system dysregulation than CA4, consistent with its critical role in postnatal neurogenesis and more robust reserve mechanisms [14].

### 3.2. CA1 Region and CA2 Region

A strikingly different pattern is observed in the CA1 and CA2 regions, which are the most vulnerable to AD [2,3]. Already at the stage of physiological aging, these regions show signs of HIF system imbalance, which appear to create a predisposition for subsequent neurodegeneration. In AD, a common feature of these regions is a sustained increase in HIF-1α (in both nuclei and cytoplasm) accompanied by a decrease in HIF-3α. Such an imbalance may have important pathogenetic consequences. First, excessive HIF-1α stimulates amyloidogenesis by increasing BACE1 transcription and β-secretase activity, as well as activating the γ-secretase complex, thereby enhancing Aβ production [3]. Second, HIF-1α modulates microglial activation, promoting chronic neuroinflammation and neurodegeneration progression [2]. The concomitant decline in HIF-3α—a negative regulator of HIF-1α [4]—removes inhibitory control and further amplifies HIF-1α-mediated effects.

The key difference between the CA1 and CA2 neuronal responses lies in the change in HIF-2α levels. In CA1 neurons in AD, HIF-2α accumulates in the cytoplasm while the nuclear pool remains unchanged. Such uncoupling may indicate impaired nuclear import of HIF-2α (possibly due to oxidative modification of importins [18]), which could negate its protective transcriptional potential despite increased synthesis. This is consistent with reports that cognitively preserved patients with amyloid pathology, by contrast, show nuclear accumulation of HIF-2α, associated with protection against tau pathology [14]. In CA2 in AD, HIF-2α decreases in both nucleus and cytoplasm compared to aging. This may indicate depletion: despite compensatory HIF-2α upregulation during aging, in AD chronic oxidative stress and amyloid toxicity suppress its expression or stability, potentially leading to reduced neuronal antioxidant defense.

### 3.3. Effect of the Vascular Component on Hippocampal Neuron Response

In the hippocampal CA1 region, HIF-1α and HIF-3α levels in AD with CAA do not differ from those in classic AD, indicating that the addition of a vascular hypoxic component does not modify the HIF isoform imbalance observed in AD in this region. This is likely explained by the maximal activation of the HIF-1α signaling pathway in AD [5]. However, HIF-2α levels in AD with CAA are significantly reduced in both the nucleus and cytoplasm compared to AD. Loss of HIF-2α in the context of combined pathology may lead to dysregulation of antioxidant defense and mitochondrial respiration [17], aggravating the neurodegeneration. Thus, in CA1, HIF-2α appears to be the most vulnerable link when a vascular component is added.

In the hippocampal CA2 region, a similar pattern is observed: HIF-1α and HIF-3α levels in AD with CAA mirror the changes characteristic of classic AD, which may reflect maximal activation of HIF-1α signaling mechanisms [5]. At the same time, HIF-2α levels are reduced not only compared to aging but also further decreased compared to AD. This suggests that severe hypoperfusion caused by CAA exacerbates HIF-2α loss, likely through enhanced oxidative stress and ischemia.

In the hippocampal CA4 region, the effect of the vascular component follows a different pattern. In AD with CAA, the nuclear level of HIF-1α does not change compared to aging or AD, whereas cytoplasmic HIF-1α decreases compared to AD, remaining at the level characteristic of physiological aging. This may indicate depletion of cytoplasmic HIF-1α, possibly due to its accelerated degradation via transcription-dependent exhaustion, a mechanism described under prolonged hypoxia conditions [13]. HIF-3α levels in AD with CAA remain reduced, as in AD, confirming the key role of HIF isoform imbalance in the hippocampus in AD, independent of the vascular component. HIF-2α levels in the cytoplasm of CA4 neurons in AD with CAA are further increased compared to aging and AD, while its nuclear level remains unchanged. This may reflect enhanced synthesis or stabilization of HIF-2α in response to more severe hypoperfusion caused by CAA. However, the unchanged nuclear pool suggests a possible impairment of nuclear translocation [18].

In the hippocampal DG, HIF-1α levels in AD with CAA are significantly lower than in AD, remaining at the level characteristic of physiological aging. The addition of the chronic vascular component of CAA leads to a reduction in HIF-1α, possibly through accelerated degradation via transcription-dependent exhaustion [13]. Unlike in CA4, this reduction in the dentate gyrus is more pronounced, affecting both cytoplasmic and nuclear fractions of the protein. Thus, while the dentate gyrus demonstrates resistance to acute ischemia [16], our data suggest that it is more vulnerable to the chronic hypoperfusion characteristic of CAA, which may be related to the high metabolic demands associated with neurogenesis. HIF-3α levels in AD with CAA fall below those in isolated AD, indicating exhaustion of the HIF-1α signaling pathway, where loss of the inhibitor is no longer reflected in protein levels. Given that HIF-1α is required for maintaining the neuronal progenitor pool [14], its reduction may lead to a contraction of this pool, contributing to cognitive decline. In contrast to HIF-1α and HIF-3α, HIF-2α levels in DG neurons in AD with CAA do not differ from values in either aging or AD, remaining stably elevated, which may provide antioxidant defense even under chronic hypoperfusion conditions.

**Figure 10:**
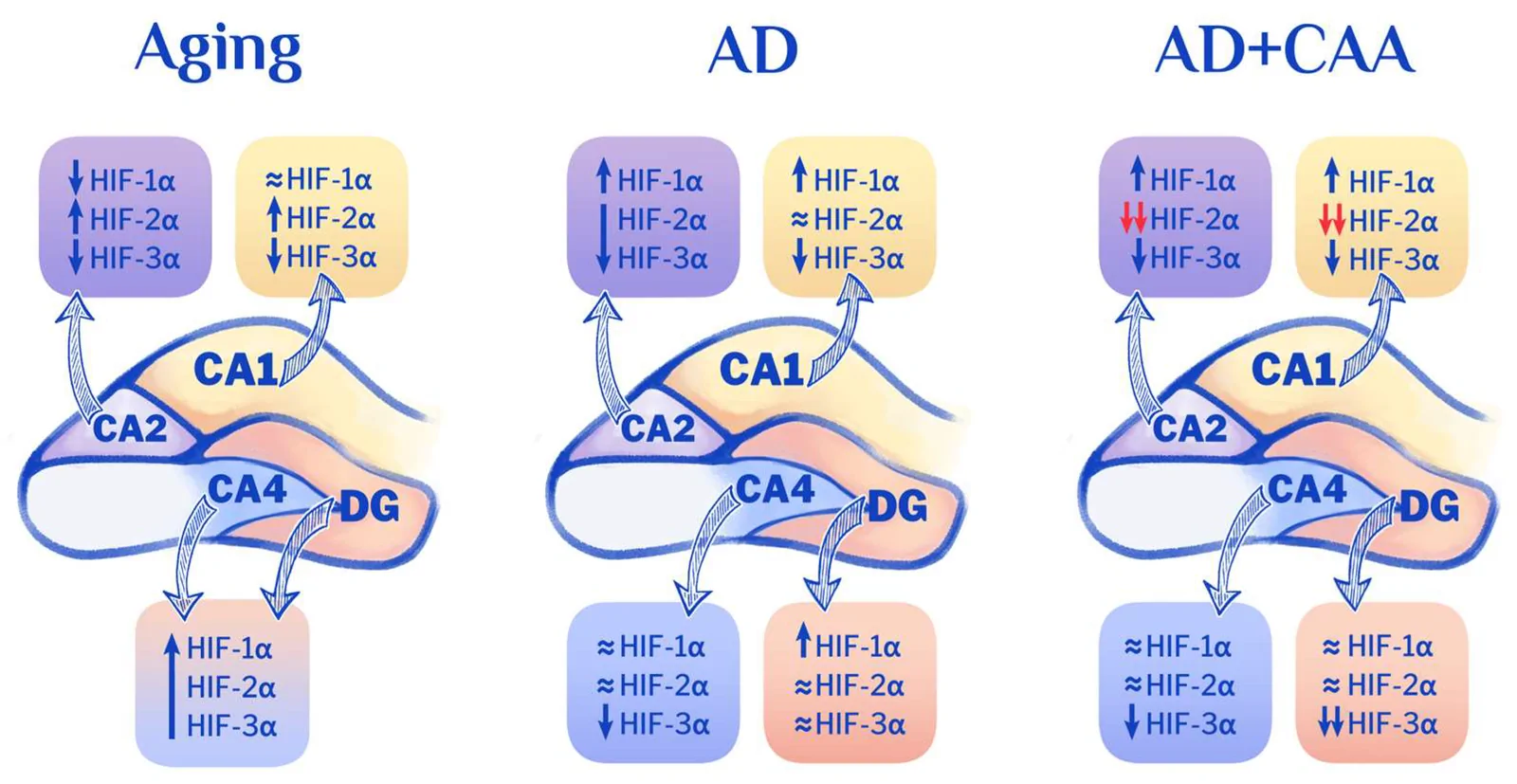
Graphical summary of HIF-1α, HIF-2α, and HIF-3α expression profiles in neurons of human hippocampal subfields (CA1, CA2, CA4, DG) across aging, Alzheimer’s disease (AD), and AD with cerebral amyloid angiopathy (CAA). Arrows indicate changes in protein nuclear expression relative to the specified comparison group: single arrows—changes versus the young group (aging panel) or versus the aging group (AD and AD + CAA panels); double arrows—changes in the AD + CAA group versus the AD group. ↑ = increase; ↓ = decrease; ≈ = no significant change.

Thus, the vascular component in CAA modifies HIF expression in a region-dependent manner: in the areas most vulnerable to neurodegeneration (CA1, CA2), it exacerbates HIF-2α deficiency (Figure 10). In resistant areas (CA4, DG), chronic hypoperfusion conversely reduces HIF-1α (likely through transcription-dependent exhaustion) while maintaining or even increasing HIF-2α. An exception is the DG, where an additional decrease in HIF-3α indicates more profound dysregulation, potentially disrupting neurogenesis. Therefore, HIF-2α emerges as a regionally sensitive isoform in the context of combined AD and CAA pathology, with its depletion correlating with regions of greater neurodegeneration. This pattern supports the hypothesis that HIF-2α loss may contribute to regional vulnerability, though causal validation requires future functional studies.

### 3.4. Anterior Cingulate Cortex

A fundamentally different pattern is observed in the anterior cingulate cortex, which, unlike hippocampal neurons, maintains a balanced regulatory response of the three HIF isoforms during aging and AD.

In physiological aging, neurons in layers 3 and 5 of the anterior cingulate cortex show a synchronous increase in all three HIF isoforms, similar to that observed in CA4 and DG. This may reflect adaptive changes to the high metabolic demands of pyramidal neurons [19], which form the core of layers 3 and 5. The high neuronal activity is associated with significant oxygen consumption, which, as has been shown, can create local oxygen deficits (functional hypoxia) even under normoxia conditions, leading to HIF-1α stabilization [20]. In addition, the age-related increase in ROS production inhibits prolyl hydroxylases, further stabilizing HIF-1α [15]. The concomitant increase in HIF-3α indicates preserved negative feedback control, aligning the anterior cingulate response with the ischemia-resistant hippocampal regions. This is consistent with evidence that preservation of the anterior cingulate cortex is a key feature of successful cognitive aging and resistance to tau pathology [21,22], suggesting that it retains a relatively intact adaptive capacity during aging.

In AD, neurons in layers 3 and 5 of the anterior cingulate cortex show a simultaneous increase in both HIF-1α and its inhibitor HIF-3α. This indicates enhanced negative regulatory control over HIF-1α even in the context of neurodegeneration, distinguishing the anterior cingulate cortex from all hippocampal regions, where HIF-3α was either decreased or remained at aging levels. Thus, the anterior cingulate cortex appears to retain the capacity to regulate the HIF system in AD. However, the role of HIF-3α in neurodegeneration is complex: in addition to inhibiting HIF-1α, its upregulation enhances oxidative stress and mitochondrial dysfunction in astrocytes through TXNIP activation [23], and also triggers the pro-inflammatory Hif3α/Rab7/TNFα/IL1β cascade, promoting neuroinflammation and β-amyloid deposition [24]. High HIF-1α levels also activate amyloidogenesis and neuroinflammation [2,3], which, together with the pro-oxidant and pro-inflammatory effects of HIF-3α, may contribute to neurodegeneration in this region.

In AD with CAA, the anterior cingulate cortex shows for the first time a distinction between the cellular responses of layers 3 and 5, which had responded uniformly during aging and in AD. In layer 3 neurons, HIF-1α and its inhibitor HIF-3α return to aging levels. This may reflect relative exhaustion of the HIF system upon addition of the chronic vascular component of CAA, possibly through accelerated degradation of HIF-1α via transcription-dependent exhaustion [13] and consequently reduced HIF-1α-dependent transcription of HIF-3α. In layer 5, by contrast, HIF-1α levels remain elevated, as in AD, indicating relative preservation of the HIF-1α signaling pathway under chronic hypoperfusion conditions. HIF-3α levels decrease compared to AD but remain above aging levels, reflecting partial attenuation of HIF-1α negative regulation. Thus, in AD with CAA, layer 5 neurons of the anterior cingulate cortex show greater resilience, which may be related to the higher metabolic reserve of large pyramidal neurons [19] and is consistent with evidence that the cerebral cortex and hippocampus respond differently to chronic hypoperfusion [25].

A key feature of anterior cingulate cortex neurons is the persistently elevated HIF-2α levels across all groups studied—aging, AD, and AD with CAA, significantly distinguishing it from the hippocampus. Such stability of HIF-2α likely provides adequate antioxidant and mitochondrial protection, maintaining neuronal energy homeostasis even under chronic hypoperfusion conditions.

### 3.5. Convergent Evidence from Experimental Models and Implications for Future Studies

The central tenet of our model—that HIF-2α is the most vulnerable link when a vascular component is added to AD pathology—finds independent support from experimental data. Van Acker et al. [26] demonstrated that neuroglobin and cytoglobin, effectors functionally analogous to the downstream HIF-2α targets (SOD2, EPO), fail to upregulate under hypoxic challenge conditions in amyloid-burdened mice, a phenomenon specifically tied to the vascular/CAA-like component of pathology. This observation parallels our finding that HIF-2α is selectively diminished in human hippocampal neurons in combined AD and CAA pathology, providing conceptual support for the proposed model across species and experimental paradigms. However, several methodological distinctions warrant careful consideration. The cited data are transcript-level, acute-hypoxia mouse findings, whereas our study provides protein-level analysis in human postmortem tissue. Given these differences in experimental design, the convergence should be framed as hypothesis-generating rather than confirmatory.

We also acknowledge that our conclusions rely primarily on IHC with a single antibody per HIF isoform. Orthogonal validation (WB, IF with independent clones, RNAscope, qPCR, LCM) would substantially strengthen the observed regional patterns. Thus, the functional interpretations proposed here remain provisional until confirmed by complementary methodologies. To address these limitations, future studies should pursue a two-pronged approach: (i) direct examination of downstream HIF-2α effectors (SOD2, EPO at protein level) in human hippocampal and cortical tissue and (ii) functional manipulation of HIF-2α in neuronal cell models—via knockdown or overexpression—under combined Aβ and hypoxic stress, with readouts including cell viability, ROS, and mitochondrial function. Such experiments would directly test whether HIF-2α status causally modulates neuronal vulnerability in the context of AD and CAA. In parallel, correlating regional HIF-2α expression with cognitive outcomes and neuroimaging markers of hypoperfusion would help establish its potential as a biomarker of regional susceptibility to combined amyloid-vascular pathology.

## 4. Materials and Methods

### 4.1. Study Setting and Tissue Samples

This study used postmortem brain tissue obtained from the Russian Center of Neurology and Neurosciences (Moscow, Russia). Postmortem tissue was obtained from deceased individuals divided into four groups:

- Group 1 (young age)—20 individuals aged 35–44 years (12 females, 8 males) with a diagnosis of sudden cardiac death resulting from acute massive blood loss or mechanical asphyxia due to severe polytrauma.
- Group 2 (aging)—20 elderly patients aged 75–99 (12 females, 8 males) years without AD, who died from pulmonary embolism following prolonged immobilization.
- Group 3 (AD without CAA)—15 deceased individuals with confirmed sporadic AD (Braak stage 3–4, Thal stages III–IV, C2 on the CERAD) aged 75–99 years (9 females, 6 males). Cause of death: pulmonary embolism.
- Group 4 (Alzheimer’s disease with cerebral amyloid angiopathy, AD + CAA)—15 patients of similar age. (9 females, 6 males) with confirmed sporadic AD (Braak stage 3–4, Thal stages III–IV, C2 on the CERAD) and CAA (2–3 points on the Vonsattel scale). Cause of death: pulmonary embolism associated with post-infarction or atherosclerotic cardiosclerosis and hypertensive disease.

### 4.2. Exclusion Criteria

Acute or previous stroke, chronic neurological and neuromuscular diseases, cardiovascular and other systemic decompensated diseases (severe insufficiency), acute infectious, infectious-allergic, and autoimmune diseases, stage III–IV malignant tumors of any location, alcoholism, and drug addiction. In addition, deceased individuals with asymptomatic hemodynamically significant atherostenosis of cerebral arteries (arterial lumen narrowing of 70% or more), as well as those with a history of stage 3 arterial hypertension, were not included in the study.

### 4.3. Tissue Processing and Histology

For microscopic analysis, blocks were excised from the hippocampal region (middle portion of the temporal lobe) and anterior cingulate cortex with dimensions: length 2–2.5 cm, width 1–1.5 cm, thickness 0.5–0.7 cm. Postmortem interval did not exceed 6 h in all cases. The fragments were fixed for 24 h in 10% neutral buffered formalin, then underwent standard laboratory processing (dehydration in alcohol, xylene clearance) and were embedded in paraffin.

### 4.4. Immunohistochemistry and Microscopy

Serial 3 μm sections were cut on a microtome, placed on positively charged slides (SuperFrost Plus, Thermo Scientific, Waltham, MA, USA), and dried overnight at 48 °C. For general morphological assessment, sections were stained with hematoxylin and eosin (H&E) and by the Nissl method. Immunohistochemical analysis was performed using a panel of four primary antibodies:

- HIF-1α (polyclonal, ThermoFisher (Waltham, MA, USA), PA1-16601, 1:200);
- HIF-2α (polyclonal, Solarbio (Beijing, China), K009522P-100UL, 1:100);
- HIF-3α (polyclonal, ThermoFisher (Waltham, MA, USA), PA1-16952, 1:200).

Antibodies were detected using the Novolink Polymer Detection System (Leica Biosystems, Nussloch, Germany), a polymer-based peroxidase visualization kit. Serial sections were cut from paraffin blocks. Each antibody was applied to a separate section using chromogenic immunohistochemistry. All serial sections were then compared.

### 4.5. Image Acquisition and Quantitative Analysis

Digital micrographs were captured using a Leica DMLB microscope (Leica Microsystems GmbH, Wetzlar, Germany) equipped with a Leica DC-300 camera (Leica Microsystems GmbH, Wetzlar, Germany). Using Leica Qwin 2.7 software (Germany); the intensity of the immunoperoxidase reaction was assessed in the nuclei and perikarya of 150 neurons per case in the CA1, CA2, CA4 and DG regions of the hippocampus and 3 and 5 layers of the anterior cingulate cortex at objective magnification 40×. Using a Wacom graphic tablet (Kazo, Japan), the outlines of neuronal bodies and nuclei were traced (excluding nuclear areas). The mean intensity of the outlined cytoplasmic areas of neurons was measured on 8-bit images (0 = white, 255 = black), with subsequent subtraction of background staining values.

### 4.6. Statistical Analysis

Statistical analysis and data visualization were performed using Statistica 13.0 and GraphPad Prism 8.0 software. For statistical analysis of morphometric data, mean values for each parameter were first calculated per case based on 150 neurons. The normality of data distribution was evaluated using the Shapiro–Wilk test. Intergroup comparisons were made using one-way ANOVA with the Tukey HSD test. A *p*-value of less than 0.05 was defined as the threshold for statistical significance.

## 5. Conclusions

In this study, we performed for the first time comprehensive comparative analysis of the distribution of three HIF isoforms (HIF-1α, HIF-2α, and HIF-3α) in neurons of various hippocampal subfields and the anterior cingulate cortex in AD and in AD with CAA. Our findings demonstrate that regional HIF isoform imbalance may underlie the selective vulnerability of different brain structures in AD. The most significant differential marker is HIF-2α: its depletion or impaired nuclear translocation was found in neurons of the most vulnerable regions, whereas its maintenance at high levels characterized regions with relative resistance. The addition of the vascular component in CAA exacerbates HIF-2α loss in vulnerable areas (CA1, CA2). The anterior cingulate cortex, by contrast, shows persistently elevated HIF-2α levels across aging, AD, and AD + CAA groups, which likely underlies its greater resilience to neurodegeneration.

## Data Availability

All the data are shown in the main manuscript.
